# Selection of Biophysical Methods for Characterisation of Membrane Proteins

**DOI:** 10.3390/ijms20102605

**Published:** 2019-05-27

**Authors:** Tristan O. C. Kwan, Rosana Reis, Giuliano Siligardi, Rohanah Hussain, Harish Cheruvara, Isabel Moraes

**Affiliations:** 1 National Physical Laboratory, Hampton Road, Teddington TW11 0LW, UK; tristan.kwan@npl.co.uk (T.O.C.K.); rosana.thevenot@npl.co.uk (R.R.); 2 Research Complex at Harwell, Rutherford Appleton Laboratory, Harwell Science and Innovation Campus, Didcot OX11 0FA, UK; harish.cheruvara@diamond.ac.uk; 3 Diamond Light Source Ltd., Harwell Science and Innovation Campus, Didcot OX11 0DE, UK; giuliano.siligardi@diamond.ac.uk (G.S.); rohanah.hussain@diamond.ac.uk (R.H.)

**Keywords:** membrane proteins, biophysics, dynamic light scattering, circular dichroism, SEC-MALS, MIR, LCP-FRAP

## Abstract

Over the years, there have been many developments and advances in the field of integral membrane protein research. As important pharmaceutical targets, it is paramount to understand the mechanisms of action that govern their structure–function relationships. However, the study of integral membrane proteins is still incredibly challenging, mostly due to their low expression and instability once extracted from the native biological membrane. Nevertheless, milligrams of pure, stable, and functional protein are always required for biochemical and structural studies. Many modern biophysical tools are available today that provide critical information regarding to the characterisation and behaviour of integral membrane proteins in solution. These biophysical approaches play an important role in both basic research and in early-stage drug discovery processes. In this review, it is not our objective to present a comprehensive list of all existing biophysical methods, but a selection of the most useful and easily applied to basic integral membrane protein research.

## 1. Introduction

Biological systems are extremely complex. Therefore, it is not surprising that their study is also complex, demanding, and expensive. This is particularly noticed when working with integral membrane proteins. Making up nearly a quarter of the human genome [1,2,3], these membrane-embedded proteins are responsible for a large number of important physiological processes, including transport, signal transducing, cell adhesion, and responses to physical/chemical external stimuli. As a result, mutations or misfolding of membrane proteins are associated with many known diseases, making them attractive therapeutic targets. In fact, it is estimated that today more than 50% of the marketed drugs target integral membrane proteins, mostly G-protein-coupled receptors (GPCRs), ion channels, and solute carrier transporters [4,5,6,7,8,9]. However, despite their popularity, many of these available drugs are associated with reduced efficiency and/or unwanted side effects. This is mostly due to the poor understanding of the biochemical behaviour of the protein target, including its interaction with the drug (drug mechanism of action). Over the years, it has become clear that identification and development of new and better drugs (whether they are NMEs (new molecular entities) or NBEs (new biological entities)) always requires an extensive understanding of the relationship between protein structure and its function [8,9,10,11]. Therefore, the study of structure–function relationships of membrane proteins has become paramount in the field of biomedical research and early drug discovery [12].

Today, biophysical methods are instrumental in providing information on protein and protein–protein complexes’ behaviour in solution, including probing protein-ligand interactions [13]. Therefore, the developments and evolution of these methods, each one fit for a purpose, have become necessary. From the structural point of view, high-resolution biophysical techniques, such as X-ray crystallography (classical approach) [14], nuclear magnetic resonance spectroscopy (NMR) [14], and neutron diffraction [15], are currently considered as “mature” and well-established methods. Major developments in single particle cryo-electron microscopy (cryo-EM) [16,17,18,19,20,21,22,23] and serial (milli- and femtosecond) crystallography [23,24,25,26,27,28,29,30,31] have recently emerged as promising tools in structure determination of challenging targets, such as protein–protein complexes and membrane proteins. Nevertheless, irrespective of the biophysical method chosen for structural studies, large quantities of high-quality protein are always needed. Unfortunately, and despite the latest advances in the field, membrane proteins are still difficult to produce and purify in such large quantities. Low expression yields, poor solubility, and protein instability once extracted from the native membrane, are just a few of the technical challenges a researcher needs to overcome [32,33,34,35,36]. In this review, we present a small selection of methods, most of which can be easily implemented in any laboratory worldwide. These approaches are very useful to evaluate the quality and behaviour of purified integral membrane proteins in solution. Furthermore, they also assist in the characterisation and assessment of protein–protein complexes and protein–ligand interactions.

One should keep in mind that an ideal biophysical method does not exist, but a range of biophysical techniques and approaches that are complementary to each other can be utilised according to the researcher’s needs.

## 2. In Situ Dynamic Light Scattering (DLS)

Dynamic light scattering (DLS), also known as photo correlation spectroscopy or quasi-elastic light scattering is a simple yet powerful method that measures and characterises particles in solution. It correlates the Brownian motion of (“spherical”) particles with their sizes. Brownian motion is merely the random movement of particles due to their encounter with the solvent molecules that surround them, the larger the particles, the slower the Brownian motion and vice versa. Brownian movement is also dependent on the medium viscosity and temperature. Thus, during DLS measurements the temperature should always be known or at least be kept constant. However, proteins or other macromolecules when in solution do not always have “spherical” shapes and are constantly solvated. Therefore, the diameter calculated from a DLS instrument is always referred to as the “hydrodynamic diameter”. This is defined as the diameter of a model sphere, also known as a hard sphere, which includes the molecule of study and any other associated molecule. The hard sphere always has the same translational diffusion average speed as the particle being measured (in our case here, the membrane protein detergent complex). Therefore, Brownian motion is related to the particle size by the translational diffusion coefficient (D_T_), given by the Stokes–Einstein equation (1) where R_h_ is the hydrodynamic diameter, k is the Boltzmann’s constant, T is the absolute temperature, η is the viscosity, and D_T_ the translational diffusion coefficient.

(1) $$R_{h}=\;\frac{kT}{3\pi \eta D_{T}}\;.$$

**Figure 1:**
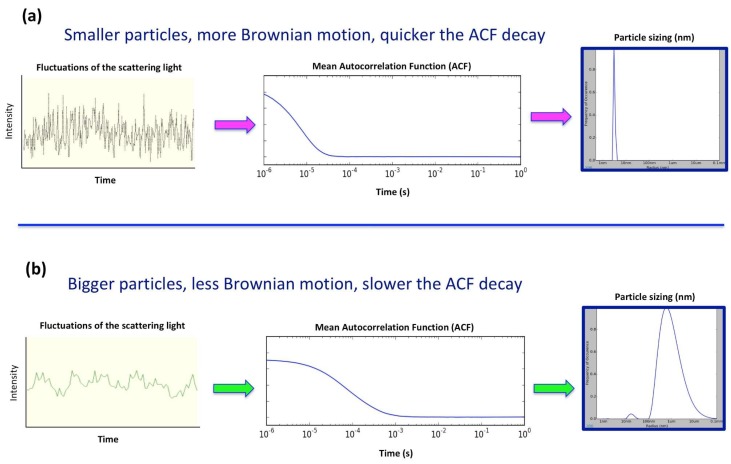
Graphic representation of the light scattering intensity fluctuation as a function of the particle size and its auto correlation function (ACF) decay for small (**a**) and large particles (**b**).

During a DLS experiment, particles in solution are illuminated by a visible and monochromatic laser light while a detector records the fraction of light scattered by the particles. The intensity of the scattered light fluctuates over time because of the endless movement of the particles (Brownian motion) resulting in constructive and destructive phases. Small particles cause intensity to fluctuate at a higher frequency than larger particles. The resultant intensity fluctuation contains information about the time-scale of the movement of the particles and can be converted into a time correlation function. The resulting decay in the correlation function is then directly associated to the motion of the particles, D_T_, and therefore its dimensions (Figure 1).

Physical principles of light scattering and DLS have been extensively described in the literature [37,38,39,40,41,42,43,44].

**Figure 2:**
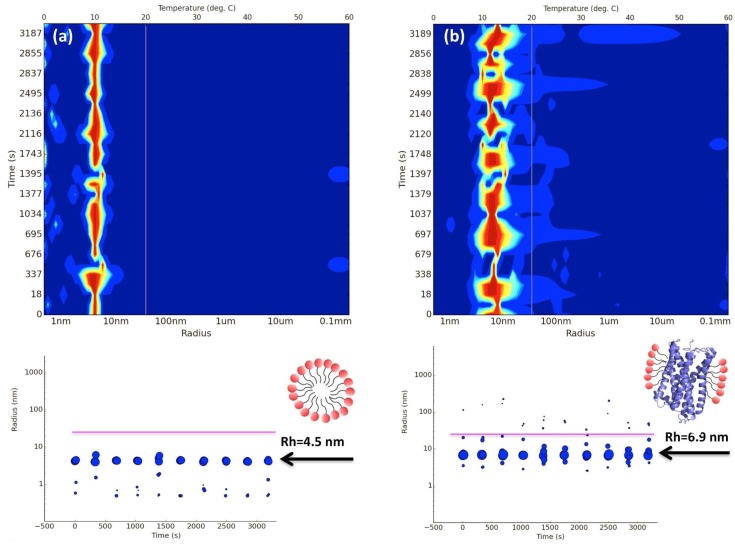
Example of protein detergent complex (PDC) identification based on “empty” micelle (**a**) and the same detergent solution in the presence of a membrane protein (**b**). The protein tested was a G-protein-coupled receptor (GPCR) solubilised and purified in 0.03% n-Dodecyl β-d-maltoside (DDM). Results from the dynamic light scattering (DLS) measurements show that hydrodynamic radius (Rh) of the pure DDM micelles is around 4.5 nm while the PDC micelle is around 6.9 nm. The in situ DLS experiments were carried out at 293 K using a SpectroLight 610 (XtalConcepts GmbH, Hamburg, Germany) instrument. 2 μL of each sample were pipetted onto a 72-well Terasaki plate and covered with paraffin oil. The experiments ran according to the hardware specifications of the instrument. The top radius distribution plots are shown in the form of signal heat maps (blue = low particle concentration, red = high particle concentration).

**Figure 3:**
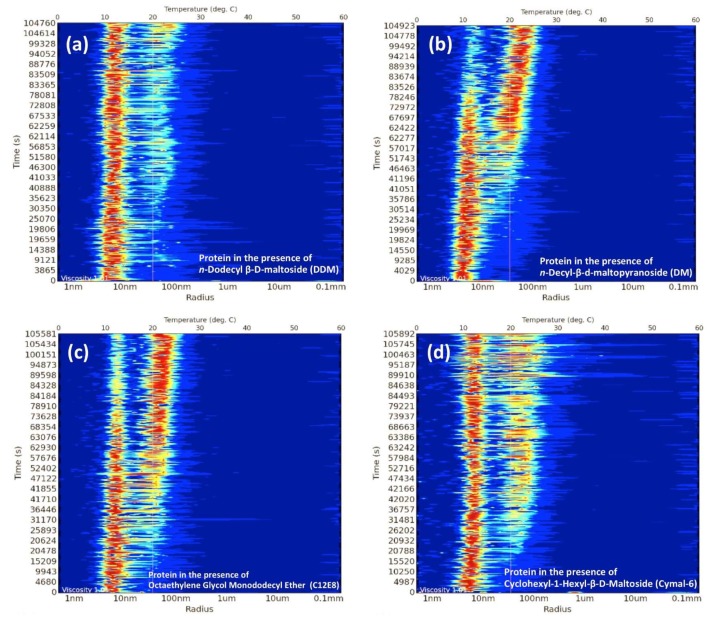
Illustration of a multi-detergent screening experiment assessing protein stability over time. Each panel corresponds to the same protein (a membrane protein transporter) in the presence of different detergents over a period of around 29 h. The size distribution plots are in form of signal heat maps (blue = low particle concentration, red = high particle concentration). The in situ DLS radius distribution panels clearly show the appearance of protein aggregates at different rate for each different detergent. The most stable sample is shown in panel (**a**). Here, the protein in the presence of n-Dodecyl β-d-maltoside (DDM) detergent takes a longer time to form larger particles (nucleation) that leads to crystal formation after 3 days. Panel (**b**–**d**) shows that the protein in the presence of those detergents starts forming large aggregates in less than 24 h, in fact the protein does not crystallise when purified in these detergents. The in situ DLS experiments were carried out at 293 K using a SpectroLight 610 (XtalConcepts GmbH, Hamburg, Germany) instrument. 2 μL of each sample were pipetted onto a 72-well Terasaki plate and covered with paraffin oil. The experiments ran according to the hardware specifications of the instrument. The protein was 10× diluted in each buffer (20 mM Tris, pH 7.5, 150 mM NaCl plus 3x critical micelle concentration (CMC) of each detergent) and left to equilibrate for 1 h. Following this, samples were centrifuged at 14,000× *g* for 20 min at 4 °C.

For many years, DLS has been considered a fast and sensitive method to analyse the behaviour of proteins in solution, in particular to probe for aggregation and stability. Today, the method is well established in most academic and industrial research laboratories covering a large variety of applications [45]. Recently, this non-invasive technique has also been used to monitor protein behaviour during the early stages of the crystallisation process [46,47,48,49,50]. As a result, DLS has emerged as a promising tool to investigate and characterise the behaviour of integral membrane proteins in solution [51,52,53]. Upon solubilisation with detergents, protein-detergent complexes (PDC) coexist with detergent free micelles and detergent monomers. However, the stability of membrane proteins in the presence of detergents is always a concern during the purification process. If detergent concentration becomes too low, membrane proteins tend to aggregate and precipitate, but on the other hand, excess of detergent may lead to denaturation or dissociation of protein complexes [36,51,54,55,56]. Therefore, great attention should always be paid to the stability of the PDC. Protein homogeneity is also very important, in particular when the protein sample is for NMR, X-ray crystallography, small angle X-ray scattering (SAXS), or single particle cryo-EM studies. Size exclusion chromatography (SEC) has been widely used as the method of choice to monitor protein quality (homogeneity and aggregation). However, the method takes a long time to run and requires large amounts of buffers and protein, and hence is considered low throughput if used as a screening tool. Recently, in situ high throughput DLS has been developed which makes use of multi-well plates (standard SBS crystallisation plates or Terasaki microbatch plates) to screen many different conditions in parallel. The method uses very low protein volumes (0.5 to 2 μL) as well as very low protein concentration (0.3 to 50 mg/mL). This new and totally automated approach has opened the doors to many capabilities in membrane protein research, such as detergent-micelle analysis (homogeneity), detergent screening for best PDC formation, protein stability over time (from hours to days), crystallisation drop nucleation/precipitation analysis (using sitting drop crystallisation plates) and the formation of liposomes [51,52]. As detergent micelles are highly uniform objects in terms of size and stoichiometry, their distribution signature can easily be used to distinguish “empty” micelles from the PDC. “Empty” detergent micelles usually show DLS peaks (or distribution signatures) that are slightly smaller and narrower than the PDC peaks. It has also been observed that DLS of membrane proteins in complex with detergents display an average particle radius size between 5 to 10 nm that should be stable over time (Figure 2). All these features combined with sample miniaturization and automation provides a fast and reliable method to screen a large number of conditions. Figure 3 shows an example of a multi-detergent screening experiment assessing protein stability over time. Size distributions given in the panel can be used to identify the best PDC signature in terms of PDC homogeneity and stability. When performing these screening experiments, it is important to note that the detergent is always added to the sample in excess and incubated for a short period of time (around 10 to 20 min) to allow for detergent exchange.

## 3. Characterisation of Membrane Proteins by Size-Exclusion Chromatography Multi-Angle Light Scattering (SEC-MALS)

Behaviour of membrane proteins in vitro is challenging and difficult to predict. Intrinsic protein instability and the use of detergents during protein solubilisation and the purification processes are just a few of the hurdles that are blamed for this [32,33,34,35,36]. It is also known that many membrane proteins in their natural cellular environment function as oligomeric complexes [57,58,59]. Therefore, it is important not only assess the quality and integrity of the PDC, but also its oligomeric state in the presence of detergent/lipid solutions before undertaking any functional, biophysical, or structural studies. Size-exclusion chromatography (SEC) has been the most common method in providing information regarding the molecular weight and homogeneity of protein samples. However, the method is based on retention volumes that are usually calibrated for globular water-soluble proteins. In fact, for membrane proteins, the relative size determined from SEC relates to the PDC. Sodium dodecyl sulphate polyacrylamide gel electrophoresis (SDS-PAGE) is another common technique used to determine molecular weight and thus validate the oligomeric state of proteins during the purification process. When working with membrane proteins however, this technique can be misleading due to different migration patterns often observed for membrane proteins [60]. Consequently, analytical determination of molecular weight and oligomeric states of membrane proteins in detergent/lipids solutions is often difficult to obtain with the laboratory tools tailored for non-membrane proteins.

Size exclusion chromatography multi-angle light scattering (SEC-MALS) is an accurate and versatile biophysical technique that is able to determine the composition, mass, and oligomeric state of membrane proteins in detergent solutions. For example, an important study has been conducted that highlights the practicality of SEC-MALS in determining the oligomeric behaviour of membrane proteins in the presence of different detergents [61]. The study shows that the oligomeric state of the *S. aureus* mechanosensitive channel (SaMscL) in vitro is dependent on the detergent used for the solubilisation/purification of the protein. In the presence of Triton X-100 and C_8_E_5_, the protein is predominantly pentameric as confirmed by SEC-MALS along with other techniques agreeing to the cross-linking studies performed in vivo. However, when solubilised in lauryldimethylamine-N-oxide (LDAO), SaMscL shows a tetrameric arrangement that is corroborated with data from analytical ultracentrifugation, SEC-MALS, and X-ray crystallography. In addition, the method can also measure the amount of “free” detergent micelles in the sample that are crucial to consider prior to crystallisation trials [62,63,64,65,66,67,68] and other assays.

**Figure 4:**
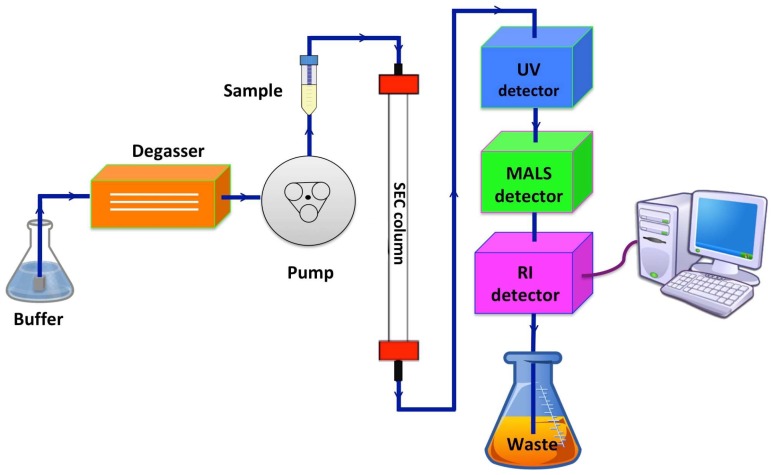
Schematic representation of a size-exclusion chromatography multi-angle light scattering (SEC-MALS) system. The buffer is de-gassed and pumped into the system. The sample is loaded into the system and carried through the size-exclusion chromatography (SEC) column where the size separation process takes place before the different components of the sample passes through the three detectors: a ultraviolet (UV) detector, a multi-angle light scattering (MALS) detector, and a refractive index (RI) detector. The detectors’ output signals are combined and analysed by the available software.

The SEC-MALS technique involves a SEC column connected in-line to ultraviolet (UV), light scattering (LS), and refractive index (RI) detectors (Figure 4). The use of a SEC column is very important since it contributes to the physical separation of aggregates, PDC, and free detergent micelles. This allows for the different mass components to be independently analysed by the detectors. Moreover, the SEC column ensures that the eluted protein sample is in the same buffer as the reference buffer (without the protein), which is crucial for an accurate determination of the three detectors’ baselines and ensures that RI fluctuations are dependent on the protein/detergents present and not by the buffer. The type of SEC column used for SEC-MALS experiments mostly depends on the sample’s size, although the most common (but not necessarily) for membrane protein work are the Superdex 200 or Superose 6 resins. It is also important to mention that certain SEC columns are reported to “shed” (release of small particles from the stationary phase), particularly if they are new. The “shedding” potentially increases the signal to noise ratio in the detectors. Hence, an extensive column wash and equilibration (bypassing the detectors) is essential before the experimental run. An on-line degasser is also recommended to ensure the stability of the detectors, thus avoiding noise increase or spikes in the output signals [62,64].

**Figure 5:**
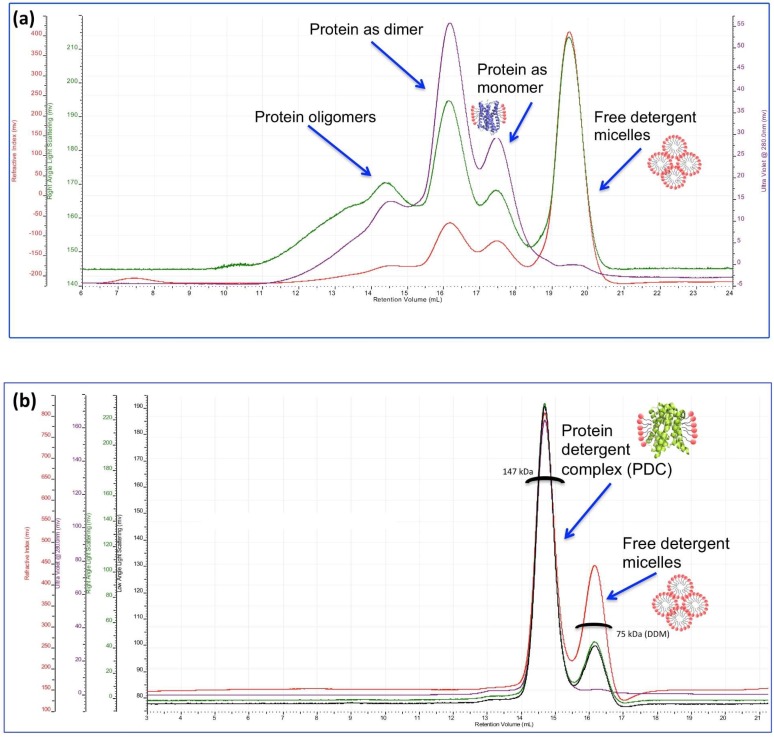
SEC-MALS analysis of two different membrane proteins. The different chromatograms correspond to the different detector signals. The trace in purple corresponds to the ultraviolet (UV) signal, green and black to the light scattering (LS) signal, and red to the refracting index (RI) signal. Panel (**a**) shows a SEC-MALS analysis of a membrane protein purified in 6-Cyclohexyl-1-Hexyl-β-d-Maltoside (Cymal-6). The chromatogram from the SEC-MALS analysis shows several peaks (detected by the different detectors) corresponding to (i) protein oligomers, (ii) protein as a dimer, (iii) monomeric protein, and (iv) free detergent micelles. Quantification of the individual peaks was not possible as they were not resolved. Nevertheless, the analysis shows that the sample has an excess of free detergent micelles and is not monodisperse. This sample would not be recommended, for example, for X-ray crystallography, cryo-electron microscopy (cryo-EM), or small angle X-ray scattering (SAXS) studies. Panel (**b**) shows a SEC-MALS analysis for the lactose permease (LacY) of *Escherichia coli* purified in 0.03% n-dodecyl-β-d-maltoside (DDM). The analysis shows a main peak that corresponds to the protein-detergent complexes (PDC) in DDM eluting at ~14.5 mL. An additional peak at the elution volume of ~16.3 mL was given by the RI and LS detectors, but not by the UV detector, as the detergent does not absorb at 280 nm. This peak corresponds to the free detergent (DDM) micelles. Calculations from the SEC-MALS analysis gave a value of 147 kDa for the PDC and approximately 75 kDa, which is the molecular mass of empty DDM micelles. In conclusion, the chromatogram shows a monodisperse LacY protein sample with no significant excess of free detergent [62]. The SEC-MALS analysis was performed using the GPC/SEC system from Malvern and the OmniSEC software from Viscotek.

In the SEC-MALS system, the UV detector is responsible for the measurement of the protein concentration based on its absorbance at 280 nm, while the LS detector measures the absolute molecular weight of the sample components based on the Rayleigh–Gans–Debye equation [61]. As the intensity of the light scattered is dependent on the size of the molecules being measured, most of the available instruments measure the scattered light at least at two different angles relative to the incident beam. The right-angle light scattering (RALS) detector measures the intensity of light scattered at 90° to the incident beam, maximizing the instrument’s signal detection for small molecules/proteins. When working with larger proteins or molecules, the intensity of the scattered light not only increases but also varies in scattering angle and, hence, the scattered intensity is also measured by a low angle light scattering (LALS) detector at 7° relative to the incident beam. Lastly, the RI detector measures the concentration of all components in the sample, including the free detergent micelles. The latter is a vital piece of information as dialysis and protein concentration (steps that might be involved in the final protein purification process) can result in an unknown amount of detergent in the sample. The value of the RI as a function of the concentration (*dn/dc*) combined with the LS data is used to calculate the absolute molecular weight of the membrane protein, its oligomeric state, and the size of the PDC (Figure 5).

The presence of detergents in membrane protein buffers has a significant effect on detector constants (K) and, in particular, the refractive index. Therefore, to avoid errors in the constants of the detectors that could propagate to the final calculations, the method involves a pre-run with a protein standard (as a calibration of the system). The best protein standard to be used should not interact with the detergent in the buffer and is of known concentration, *dn/dc*, molecular weight, and absorbance value at 280 nm. Although there are reports of bovine serum albumin (BSA) interacting with certain detergents, BSA has been used successfully as a protein standard in membrane protein SEC-MALS runs.

Data processing and SEC-MALS analysis vary among instrument manufactures. However, the “three-detector” and the “ASTRA” methods are the most used [65,66,67,68]. John S. Philo and co-workers were one of the first groups to develop the SEC-MALS approach for protein analysis. As the method is based on direct readings from the UV, LS, and IR detectors, they have described it as the “three-detector method” [66].

The name “ASTRA method” comes as result of the commercial software package ASTRA, from Wyatt technologies [67], using the analysis approach described by Equation (3). Whilst the three-detector method does not require any previous information on the *dn/dc* value of the protein or detergent, the same is not true for the ASTRA method. On the other hand, the three-detector approach cannot be used if the detergent absorbs at 280 nm, but can be overcome when using the ASTRA approach (Equations (2)–(3)).

(2) $$M_{W,\;\;protein}=\;\frac{LS*UV_{280}}{K*\;A_{280,\;\;protein}*{\left(RI\right)}^{2}}.$$

Equation (2) is used by the three-detector method to calculate the average molecular weight of the protein (M_W, protein_) in the PDC, where LS is the light scattered, UV_280_ is the absorption at 280 nm given by the UV detector, K is the constant determined during the calibration of the system based on the optical properties of the sample, A_280_ is the protein UV absorption at 280 nm that can be theoretical determined from the number of Trp and Tyr residues [69], and the RI is the refractive index [65].

(3) $$M_{W,\;\;protein}=\;\frac{RI}{UV_{280,\;}}*\;\left[\left(\frac{1}{1+\delta }\right)A_{280,protein}+\left(\frac{\delta }{1+\delta }\right)\;A_{280,\;detergent}\right],$$

where δ can be taken from

(4) (dndc)PDC= [(11+δ)(dndc)protein+ (δ1+δ)(dndc)detergent].

Equation (3) is used by the ASTRA method to calculate the average molecular weight of the protein (M_W, protein_) in the PDC, where RI is the refractive index, UV_280_ the absorption at 280 nm given by the UV detector, and δ is the amount of detergent in the micelle. The *dn/dc* values of the protein and detergent are available from the literature, or otherwise need firstly to be experimentally calculated [65].

Although SEC-MALS is a low throughput technique with a few limitations—such as that (i) it separates proteins only by size and (ii) quantitative analysis can only be done for well-resolved peaks (i.e., if the PDC or other particle in solution have a similar size to the detergent free micelles, the data cannot be properly analysed)—it remains considered as a powerful method in membrane protein research.

Recently, SEC-MALS has also been used to characterise mass, aggregation, and particle size distribution of poly(styrene-co-maleic acid) lipid particles (SMALPs) and poly(styrene-co-maleimide) lipid particles (SMILPs) nanodiscs. Nanodisc technology has become a widely used membrane mimetic for functional assays in vitro, and single particle cryo-EM studies [70,71,72].

## 4. Circular Dichroism (CD)/Synchrotron Radiation Circular Dichroism (SRCD) of Membrane Proteins

Circular dichroism (CD) is the spectroscopic technique of choice to study the conformational behaviour of membrane proteins in solution as a function of environment, such as temperature, solvent composition, detergents, membrane mimics, ionic strength, precipitants, and ligand binding interactions. For proteins, CD in the far UV region (180–250 nm) is sensitive to the backbone folding [73], from which the content of secondary structure can be estimated, in particular, the number and average length of the α-helices and β-strands conformations [74,75]. In the near UV region (250–330 nm), CD spectroscopy can be used to characterise the local environment of aromatic amino acid residues (Trp, Tyr, and Phe) and the dihedral angle of disulphide bonds [76,77]. The aromatic chromophores are often located at the binding interfaces or binding sites and can be conveniently used as natural probes to detect ligand interactions qualitatively and quantitatively [78,79]. CD spectroscopy is also very effective in detecting the binding of achiral drugs for which the induced CD (ICD) of the bound species is promptly observed. For achiral ligands with electronic transitions greater than 320 nm, such as conjugated aromatic rings or flavonoid type of chromophores, they will be the only component showing CD features as the protein and the unbound ligand will be devoid of any signal. In this case, CD spectroscopy is very suited to screen unambiguously protein-ligand binding interactions without immobilising or labelling any of the components, hence being free of positive or negative false results, unlike the surface plasmon resonance (SPR) and fluorescence when using fluorophore labelling [76].

The golden rules to measure good CD spectra can be summarised as follows:

(i) To measure CD spectra in the far-UV region (180–250 nm) characteristic of the protein folding, prepare the solution of about 0.4–0.5 mg/mL of protein and use a 0.02 cm cuvette cell pathlength. In the near-UV region (250–320 nm), the measurements tend to be conducted in a 1 cm pathlength cell, and the protein concentration is calculated from extinction coefficients from aromatic side-chain and Cys residues to obtain [69] UV absorption of about 1. In both cases, the detector HV should not exceed the recommended manufacturer limit voltage that for the majority of instruments is equivalent to 600 V.

(ii) The UV transparency of the solvent is very important, as it will dictate what to use in terms of protein concentration and cell pathlength in order not to exceed the overall absorption of about 1. A chloride ion starts to absorb light at about 230 nm, hence, to obtain a spectrum of good quality at least in the 190–250 nm region, the chloride ion concentration has to be contained to about 30 mM. However, if a higher concentration cannot be avoided, a narrow pathlength of 0.01 cm or 0.005 cm has to be used. For lower pathlengths, a demountable cuvette with 20 or 10 μm can be used, enabling measurements of up to 300–500 mM NaCl or KCl solutions. The narrower pathlength will decrease the absorption of the buffer, but equally the CD of the protein. For this reason, the concentration of the protein has to be increased accordingly following the Beer-Lambert Law. Decreasing the pathlength by 10 times will require the protein concentration to be increased by 10 times. Similarly, this approach has to be applied for any other added excipient, such as detergent and lipid mixture or any other chemical agent.

The CD of a protein is obtained by subtracting the CD of the solvent, the so-called baseline, from that of the measured protein; it is essential, therefore, that the CD of the baseline is stable as a function of time.

For membrane proteins solubilised in membrane-like environments, the CD measurements can be more difficult than those of aqueous protein solutions [80]. This is because the CD spectrum of the membrane-like molecules in the absence of protein is not as stable as those of aqueous buffers requiring an equilibration time after the protein solution is injected into the cuvette cell for spectroscopic measurements. This is likely due to a perturbation of the membrane vesicle size that requires time to stabilize. Such an equilibration time may vary from protein to protein, and the membrane-like type of detergent is required to be measured. This can be easily done by scanning repeated consecutive CD spectra until at least two superimposed spectra are observed. The equilibration time calculated by multiplying the number of scans by the scan time applied to every measurement will secure a reproducible spectrum free of artefacts that otherwise would lead to misinterpretations. This approach was developed in the study of FsrC, a membrane protein histidine kinase [81,82]. For membrane proteins that can be produced only in very small quantities, the conformational study in solution requires the use of very small aperture long pathlength cuvette cells. Unlike with conventional CD instruments, the high flux collimated microbeam of Diamond B23 beamline for synchrotron radiation circular dichroism (SRCD) (https://www.diamond.ac.uk/Instruments/Soft-Condensed-Matter/B23.html) enables the measurements in such a small aperture long pathlength cuvette cell to be easily performed.

Studies of the GBAP and Siamycin I in the presence of ligands were successfully conducted at the SRCD B23 beamline (highly collimated microbeam) at Diamond Light Source, using the small aperture 1 cm pathlength cuvette [82,83,84,85].

**Figure 6:**
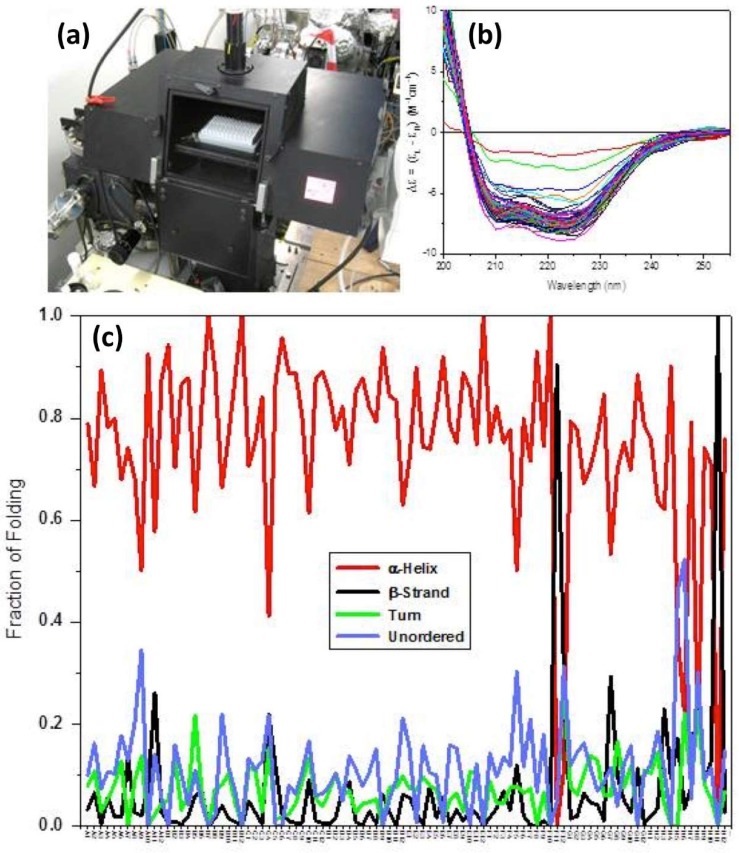
Synchrotron radiation circular dichroism (SRCD) measurements of the LacY transporter using the B23 beamline at Diamond (https://www.diamond.ac.uk/Instruments/Soft-Condensed-Matter/B23.html). (**a**) Picture of the end station at B23 beamline that enables high-throughput screening. It has been designed to accommodate the 96-well multiplates made of fused quartz (Suprasil, Hellma); (**b**) SRCD spectra of the bacterial transporter LacY. Each of the 96 distinct solutions of the MemGold2 (Molecular Dimensions) screen contained about 15 μL of 0.5 mg/mL of LacY. Measurements were done using a pathlength cell of 0.02 cm; (**c**) Percentage of α-helical content (and other secondary structure features) across the 96 MemGold2 crystallisation solutions.

Another advantage of using B23 beamline for SRCD is the unique capability of measuring in a high-throughput CD (HTCD) manner. Conformational behaviour of membrane proteins induced under different environmental conditions can significantly affect protein function including drug-binding affinity. Therefore, high-throughput screening of many buffer conditions, including crystallisation buffers and a variety of ligands, using SRCD offers a significant advantage compared to benchtop CD [86]. For example, HTCD in the far-UV region of the bacterial transporter LacY measured in a 96-cell plate revealed that only four buffer mixtures out of the initial MemGold2 98 screen formulations (www.moleculardimensions.com/products/4234-MemGold2), induced 100% α-helical content, consistent with the crystallographic structure [87]. The rest of the formulations reduced the α-helical content of the LacY transporter to 60–90% (Figure 6). These HTCD measurements in buffers with high salt and precipitant content in the far-UV region are unattainable when using benchtop CD instruments, unless they are conducted on a single cuvette basis. However, using a single cuvette basis for measurements of 96 different buffer conditions is time-consuming and labour-intensive. Hence, HTCD screening using synchrotron radiation can be used as a powerful tool in X-ray crystallography studies, including fragment-based drug design from which new potential lead compounds can be derived. This is particularly important when working with demanding targets, such as membrane proteins.

## 5. Fluorescence Dye-Based Differential Scanning Fluorimetry (DSF) Assay

As already mentioned in this review, the stability of membrane proteins remains a major bottleneck when undertaking biochemical and structural studies. During the solubilisation process, the native lipid bilayer is lost. Here, the natural lipids that used to cover the peripheral hydrophobic regions of the membrane proteins are replaced by detergent (surfactant) molecules, forming water-soluble protein-detergent complexes (PDCs). However, although currently there is a large variety of commercially available detergents, finding the best detergent that guarantees stability and functionality of the purified membrane protein, and at the same time is compatible with the downstream work plan (e.g., biochemical and biophysical assays, NMR, CD, or crystallographic studies) is still is a long and empirical process. For example, while short chain detergents such as octylglucosides are known to be more denaturing, detergents with longer chains tend to engulf proteins and therefore are not recommended for crystallisation trials or other biophysical methods [88,89]. On the other hand, detergents such as cholesteryl hemisuccinate (CHS), when used as additives, can significantly improve PDC stability. This has been particularly noticed when working with GPCRs and eukaryotic membrane proteins [90,91,92]. However, detergents are not the only factor that influences integral membrane protein folding and stability. Variation in the buffer composition, including pH, salt concentration, the addition of additives, or even the introduction of mutations/truncations, are just a few of other elements that can also influence PDC stability [88,93,94,95,96,97]. Today, a variety of methods to assess the stability of soluble and membrane proteins in solution are available. However, many of them require large quantities of protein, require expensive equipment, or are rather low-throughput.

The fluorescence dye-based differential scanning fluorimetry (DSF) method, also known as protein thermal-shift assay or ThermoFluor, is a simple yet powerful tool that was initially developed to screen protein stability in the presence of small ligands (fragments) in early drug discovery platforms [98,99,100]. Today, it is a well-established approach to assess the stability of soluble [101,102,103,104] and membrane proteins [105,106,107,108,109] in the presence of different buffer formulations, detergents, and small molecules.

**Figure 7:**
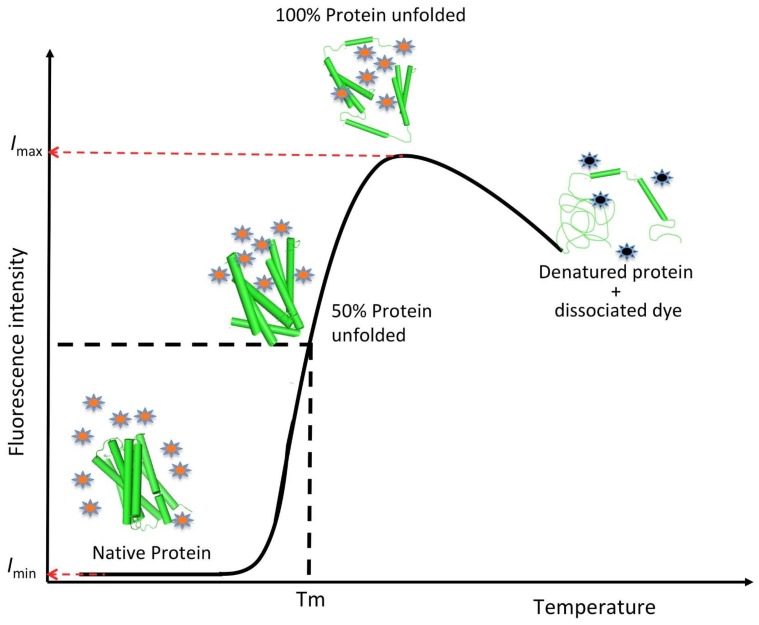
Graphic representation of a typical melting curve obtained during a differential scanning fluorimetry (DSF) experiment in the presence of a fluorescent dye. As the temperature increases, the protein unfolds and its intrinsic region becomes solvent-exposed, therefore, reacting with the fluorescent dye. The fluorescence intensity increases with the number of dye molecules binding the protein (in specific regions depending of the dye). The fluorescence intensity starts dropping with the protein denaturation and aggregation. The value of T_m_ is calculated using Equation (5), where *I_max_* and *I_min_* correspond to the lower and upper limits of the curve, respectively.

The DSF method is based on the relationship between protein stability and its Gibbs free energy of unfolding (ΔG_u_). In other words, as temperature increases, protein stability decreases, and it starts unfolding. When the amount of unfolded protein equals the amount of folded protein, the value of ΔG_u_ becomes zero and the system has reached what is known as the “melting point” temperature (T_m_). In the DSF method, the fluorescence intensity is plotted as a function of the temperature. As a result, a sigmoidal curve is generated where the inflection point of the curve is the T_m_ value. The inflection point (or T_m_ value) is easily calculated using a Boltzamann equation:

(5) $$Y\left(x\right)=\;I_{max}+\frac{\left(I_{min}\;-\;I_{max}\right)}{1+e^{\left(\frac{T_{m}-T\left(x\right)}{a}\right)}},$$

where, *I_max_* and *I_min_* are the maximum and minimum fluorescence intensity values, respectively, and *a* is the slope of the curve at the point T_m_ [102], (Figure 7).

**Figure 8:**
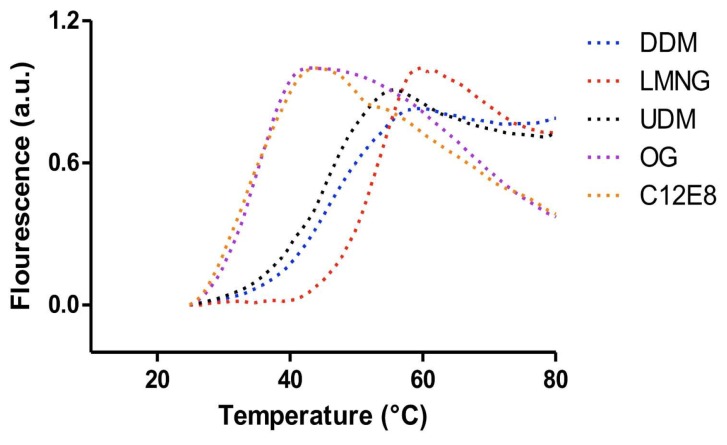
Thermal stability profiles of a membrane protein in the presence of different detergents using the thiol-specific fluorochrome N-[4-(7-diethylamino-4-methyl-3-coumarinyl)phenyl] maleimide (CPM) dye assay. The calculated melting temperatures (T_m_) were determined by fitting the curves to a Boltzmann sigmoidal (Equation (2)). The calculated values are as follows: 45.7 °C in the presence of n-Dodecyl β-d-maltoside (DDM); 50.9 °C in the presence of lauryl maltose neopentyl glycol (LMNG); 43.4 °C in the presence of n-Undecyl-β-d-Maltopyranoside (UDM); 32.2 °C in the presence of n-Octyl-β-d-Glucoside (OG); and 34.3 °C in the presence of dodecyl octaethylene glycol ether (C_12_E_8_).

An alternative representation to the sigmoidal curve is to use the negative first derivative of the experimental data (-(dRFU)/dT) to calculate the T_m_. In this case, the apex of the negative curve gives the value of the T_m_. A positive shift in the T_m_ suggests stabilisation of the protein, while a negative shift in the Tm suggests destabilisation. Hence, the variation of the T_m_ (ΔT_m_) of the protein in different buffers, detergents, and/or in the presence of different ligands gives a good estimate of the protein stability (Figure 8).

On the basis of the principle that an increase in the protein T_m_, due to ligand binding, is in general dependent of the ligand concentration and proportional to the ligand affinity, the method can also be used to estimate ligand-binding affinities (K_d_) [110].

Because DSF is relatively inexpensive, fast, and easy to set up, it has rapidly become popular among academic and industrial structural biologists to probe the best conditions (buffers, salt, substrates, inhibitors, or other small molecules) that could enhance protein crystallisation. In the DSF method, the protein of interest is diluted in a range of different buffer conditions (~1–15 μg of protein per condition) in the presence of a fluorescent dye. As the technique has the advantage of only requiring a real-time PCR (RT-PCR) machine that has the correct fluorescent filter with respect to the fluorescent dye being used, the mixture conditions are typically set up in small volumes using qPCR plates. The speed of the temperature ramp usually set up for the assay is about 1 °C min^−1^. The most commonly used fluorescent dye when working with soluble proteins is SYPRO orange, as it has high sensitivity, low interference with small molecules, and a relatively high excitation (~480 nm) and emission (~569 nm) wavelength (typically available in all RT-PCR machines). As the protein unfolds with the increase in temperature, SYPRO orange binds nonspecifically to the exposed intrinsic hydrophobic regions of the protein, resulting in an increase in fluorescence intensity. Once the protein starts to denature and aggregate as a result of increase in temperature, the fluorescence intensity decays. However, when working with membrane proteins, SYPRO orange is not a suitable dye, as it binds to detergents and lipids in the existing PDC and thereby increases the fluorescence background. To overcome this problem, a fluorescence-based thermal stability assay based on highly reactive thiol-specific fluorochrome N-[4-(7-diethylamino-4-methyl-3-coumarinyl)phenyl] maleimide (CPM) has been introduced [107,108,109]. The CPM dye has a high preference for thiols rather than for nucleophiles, which preferentially and specifically react to cysteine side chains. As the PDC unfolds because of the temperature increase, cysteine residues in the core of the protein are exposed and react with the CPM molecules. The only limitations of the method when using the CPM dye are (i) the protein in study should have cysteine residues in the transmembrane domains, and (ii) the PCR instrument must have a special filter for the excitation and emission wavelengths of the CPM dye, typically around 387 nm and 463 nm, respectively (commercially available RT-PCR machines usually do not come with these filters unless requested). The use of an alternative thiol-reactive dye, BODIPY FL-l-cysteine, has recently been reported [111]. BODIPY FL-l-cysteine (excitation 505 nm/emission 513 nm) was used to probe the stabilization of CXCR2 upon binding with different low molecular weight ligands. It is claimed that the advantage of the BODIPY FL-l-cysteine over the CPM dye is its ability to reduce compound autofluorescence artefacts, thereby reducing the number of false positive/negative results.

Finally, recent advancements in label-free DSF (nanoDSF) have also proven to be a fast and reliable approach to analyse protein stability in solution. The method is based on the changes of tryptophan fluorescence intensity upon protein unfolding (as tryptophan fluorescence is strongly related to its chemical environment). In addition, the technology is also able to measure tryptophan fluorescence intensity at two different wavelengths (330 and 350 nm). The ratio of the tryptophan fluorescence at 350 and 330 nm produces better data to calculate the thermal unfolding transition midpoint (T_m_), compared to single wavelength detection (calculations are not possible if a single wavelength data set is not good enough). The method is applicable to both soluble and membrane proteins [112,113,114]. Recently, the application of the label-free DSF to proteins that are in lipid cubic phase (LCP) also seem to be a promising tool for screening screen membrane protein behaviour and the stability in mesophase prior crystallisation trials [115].

## 6. Mid-Infrared Spectroscopy (MIR)

Although there are many methods to determine protein concentration in solution [116,117], the most popular approach is by ultraviolet (UV) spectroscopy. In this method, quantification of protein concentration is achieved by measuring the absorbance of the purified protein at 280 nm, also known as the “A280 method”. The protein absorbance is based on the property that certain aromatic amino acid residues (mostly tryptophan and tyrosine) have in absorbing UV light at 280 nm. If the extinction coefficient for the protein in study is known, the relationship between absorbance and concentration is given by the famous Beer–Lambert Law [118,119]:

(6) A= ε∗l∗c

Equation (6) is the Beer–Lambert equation, which states that absorbance is proportional to the concentration where *A* is the protein absorbance, ε is the molar extinction coefficient (mol^−1^ L cm^−1^), *l* is the cell pathlength (cm), and *c* the protein concentration (mol L^−1^). This simple method is mostly performed in laboratories by instruments that are simple to operate, fast, and able to do the measurements at low micro-litre volumes without requiring sample dilution [120], all contributing to the popularity of the method. However, the concentration calculated will not be absolute, as the ratio of tryptophan and tyrosine residues differs from protein to protein, and most of the time the molar extinction coefficient used is theoretical (calculated from the sequence). This technique becomes less reliable when calculating membrane protein concentrations because of the presence of lipids and detergents, which often interfere with the absorption at 280 nm. Furthermore, and unfortunately, this technique cannot be utilized to calculate the amount of lipids or detergent in the sample, which is of great importance in membrane protein research. Techniques such as SEC-MALS, described earlier in this review, can provide necessary information on the relative amounts of protein and detergent/lipid in the sample. However, performing this experiment may not always be possible, given the difficulties in obtaining high yields of integral membrane proteins in addition to the low throughput side of the method when screening buffers.

Recently, an infrared (IR) based protein quantification method has been proposed, which has an added value of quantifying detergent and lipids in the sample [121]. As IR radiation excites vibrational transitions of a molecule, IR spectroscopy has become a powerful tool in the detection of biomolecules, such as proteins, lipids, carbohydrates, and nucleic acids [122,123,124,125,126,127].

**Figure 9:**
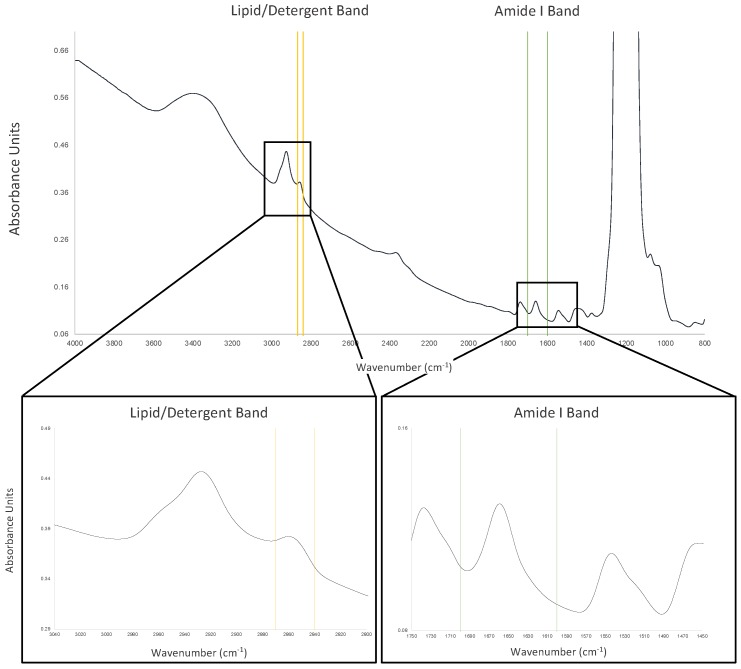
Mid-infrared spectrum of a membrane protein in n-Dodecyl-β-d-Maltopyranoside (DDM). Measurements were performed using the direct detect spectrometer (EMD Millipore). The protein sample (2 µL) was blotted onto a hydrophilic polytetrafluoroethylene (PTFE) membrane and dried for 30 s using the built-in heater/fan system before measuring the MIR spectrum. The spectrum is plotted against the inverse of the wavelength (the wavenumber). Yellow represents the aliphatic C–H stretching region of DDM between 2870 and 2840 cm^−1^. Green represents the amide I region between 1700 and 1600 cm^−1^. Note the strong signal between 1300 and 1100 cm^−1^ due to the PTFE membrane [121].

In the mid-infrared (MIR) spectrum (4000–400 cm^−1^) nine specific absorption bands, known as amide bands (amide band A, B, I, II…VII), are present. These bands are associated with different vibrational modes of the amide functional groups in biomolecules. In other words, the wavelength at which MIR radiation is absorbed by the different functional groups in the molecules, such as proteins and lipids, is a distinctive “fingerprint” for each particular molecule type. For example, proteins display the amide I and II bands at approximately 1650 cm^−1^ and about 1540 cm^−1^ due to stretching and bending of C=O and N–H bonds, respectively. In the case of lipids, because of the difference in chemical composition, absorption is observed in many different regions of the IR spectrum. However, the most characteristic peaks are the absorptions at about 1740 cm^−1^ assigned to ester C=O stretch, at 2852–2920 cm^−1^ for symmetric/asymmetric CH2 stretch, and at about 1235 cm^−1^ for phosphate stretching [121,125,126]. Most detergents, because of their chemical nature, show MIR profiles similar to those for lipids (Figure 9). 

The integration of these bands following the Beer–Lambert Law enables the determination of proteins and lipid/detergent concentration of the purified sample [121]. This provides important information regarding the sample composition and its structural properties [126] that are paramount in the study of structure–function relationships of soluble and membrane proteins [125]. Furthermore, time-resolve MIR can provide important information regarding dynamic behaviour over time of the different molecule species present in the sample.

The advantage of the MIR-based analysis compared with the absorption method at 280 nm is that it is much less dependent on the amino acid composition and it is possible to quantify lipids/detergents in the sample. The only disadvantage of the method is the overlap between water absorption and the amide I band. However, most of the instruments today are able to do water/buffer subtraction [121].

## 7. Lipidic Cubic Phase Fluorescence Recovery After Photobleaching (LCP-FRAP)

Fluorescence recovery after photobleaching (FRAP) is a valuable tool for assessing integral membrane protein behaviour (diffusion) in the lipidic cubic phase (LCP), liposomes, and sponge phases [128].

Crystallisation in LCP was first introduced in the late 1990s by Landau and Rosenbush [129]. Today, more than 500 structures in the Protein Data Bank have been solved using this approach (https://blanco.biomol.uci.edu/mpstruc/). With the advent of serial crystallography and the development of viscous extruders (also known as “LCP injectors”) [130,131,132,133], crystallisation in LCP has become more popular and almost vital in the field of time-resolved studies [27].

As a general rule, LCP is formed spontaneously by mixing the chosen lipid (usually the monoolein lipid, but not necessarily) with the protein–detergent complex solution at certain ratios and temperatures [62,129]. Structurally, LCP is a complex three-dimensional network of a bicontinuous lipid bilayer and two separated water channels that mimic the natural biological membranes [134,135]. For the monoolein/water system, the Pn3m phase has proven to be the most suitable for the crystallisation of membrane proteins. However, in contrast to crystallisation trials in aqueous solutions, where the proteins are free to move, common precipitants often induce restrictions on protein diffusion in LCP. In this scenario, LCP-FRAP serves as a simple pre-crystallisation tool for a faster identification of conditions that favour the diffusion of protein and its stability within the LCP. Although, parameters, such as protein concentration and temperature, are also important for positive crystallisation outcomes, LCP-FRAP is able to assist in the identification of best host lipid, best protein constructs, and ligands that may not promote diffusion of the protein in LCP. Commercially available LCP-FRAP instruments, with an automated and high throughput setup, allow for the screening of hundreds of conditions in a few hours using only microgram quantities of protein (working protein concentration between 1 to 5 mg/mL).

**Figure 10:**
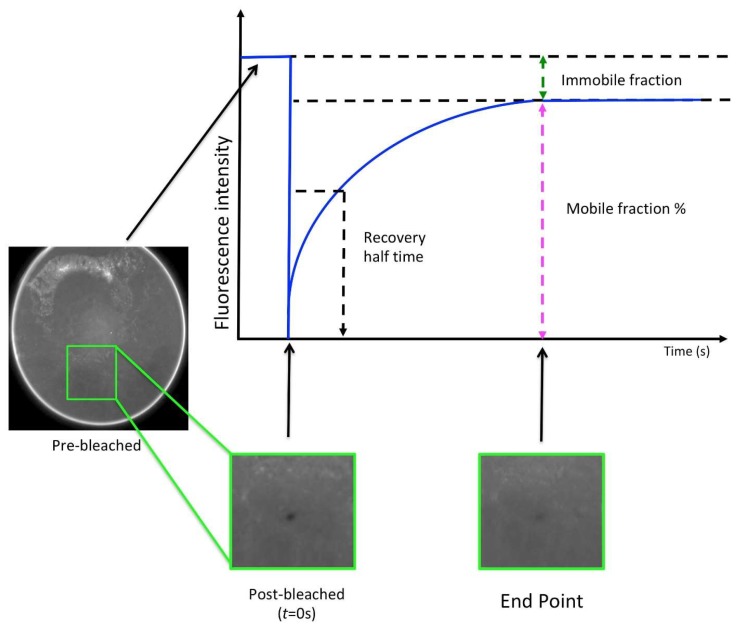
Representation of the fluorescence recovery after photobleaching (FRAP) recovery curve. The diffusion rate of a protein in LCP can be determined by FRAP, which measures the amount of time required for the fluorescence intensity of a tagged protein to diffuse out from the bleached area. The diffusion rate is given by the recovery curve and proportional to slope of the curve at the starting point (t = 0 sec) [137,138].

The method first requires the labelling of the interested protein. Usually Cy3 (5, 5′-disulfato-1′-ethyl-3, 3, 3, 3- tetramethylindocarbocyanine) dye is used. However, two variations of the Cy3 dye are commercially available. The Cy3 mono-maleimide reacts with free sulfhydryl groups of the cysteine residues, while the Cy3-mono N-hydroxylsuccinimidyl (NHS) ester reacts with free amino groups such as N-terminus and lysine residues. In the latter case, tris-based buffers are not recommended as the amine group of the buffer also reacts with the dye. Therefore, attention must be taken in choosing the one that best suits the protein and buffer conditions. After the labelling, the unbounded dye must always be removed to avoid overloads during the LCP-FRAP measurements, usually done through SEC. The LCP plates set up with the labelled protein in general are incubated at 20 °C between 8 to 15 h (dependent of the protein target) prior to FRAP in order to allow the LCP to stabilise. The FRAP measurement process occurs in four steps: (a) A sample image is taken before bleaching as the baseline fluorescence intensity; (b) The sample is photo bleached by a laser; (c) The post-bleaching image is taken as the molecules diffuse in and out, thereby diminishing the bleached spot; and (d) Normalised fluorescence intensity vs. time is analysed and plotted to get the mobile fraction and the diffusion rate (Figure 10). Finally, the mobility fraction rate can be calculated and scored, helping to determine whether the conditions are favourable or not for crystallisation [136,137,138].

## 8. Summary and Perspectives

Over the recent years, traditional biophysical methods have been combined with new developments, including improvements on detection sensitivity (signal-to-noise-ratio), low sample volume usage, and high throughput approaches, opening several new opportunities in the field of protein science. Today, these methods provide a large variety of measurements that are crucial in delivering important and complementary information to that obtained from conventional biochemical and cellular assays. Hence, it is not surprising that many of these biophysical methods are now being introduced in membrane protein laboratory workflows and are fully integrated throughout early drug discovery platforms as primary and secondary screening tools.

Given the therapeutic importance and the complexity of integral membrane proteins, structural and biochemical data are essential. However, the challenges associated with the structure determination and characterisation of integral membrane proteins are still large in number. Here, we have provided a brief overview of the most useful and easily applied biophysical methods to investigate the behaviour of integral membrane proteins in solution (in the presence of detergents/lipids). These methods provide qualitative and quantitative information on the protein in study and its interaction with small ligands, substrates, ions, and other protein counterparts. In addition, a few of these techniques also have the advantage of allowing measurements over periods of time that are fundamental to the studies of protein dynamics. The choice of method to use is mostly dependent on the existence of the instrument in the home laboratory, and on the amount of sample available. However, it is advisable to be familiar with several methods, including their advantages and disadvantages (Table 1).

The future is bright for the membrane protein research community. Many membrane protein structures have now been solved revealing the first glimpses into their mechanisms of action. Rapid developments in instrumentation and methodologies, such as the methods presented here, combined with multidisciplinary approaches will overcome many of the challenges and push membrane protein research even further. Ultimately, the goal will be a critical understanding of the cellular mechanisms that will facilitate therapeutic approaches.

## Figures and Tables

**Table 1:** Strengths and limitations of the biophysical methods presented in this review.

Strengths	Limitations	Amount of Sample Required
**In Situ Dynamic Light Scattering (DLS)**		
Simple and fast to set up Direct determination of aggregation Direct determination oligomerisation High throughput Very low protein consumption Temperature range between 4 to 40 °C Measurements over periods of time	Introduction of air bubbles affects the readings Strongly absorbing particles may not produce a good scattering signal Mixtures of particles with different optical properties will not be normally measured	Minimum: 0.3 to 2.0 mg/mL. Sample volume of 0.5–2 μL per well
**Size-Exclusion Chromatography Multi-Angle Light Scattering (SEC-MALS)**		
Direct determination of aggregation Direct determination oligomerisation Able to determine composition Able to quantify the amount of detergent in the sample Able to calculate the average molecular weight of the protein in the PDC	Low throughput Large volumes of buffer Long time to equilibrate and stabilise detectors baselines Separates proteins only by size Quantitative analysis can only be done for well resolved peaks	Minimum: 0.5 to 2.0 mg/mL. Requires a sample volume of ~160 μL
**Circular Dichroism (CD)/Synchrotron Radiation Circular Dichroism (SRCD)**		
Simple and relatively fast to set up Uses relatively low concentration and amounts of sample SRCD can operate in high throughput mode Direct determination of protein secondary structure Direct determination of protein folding/unfolding and conformational changes Direct determination of protein dynamics Able to kinetic and thermodynamics measurements	Limited to buffers that do not strongly absorb in the Far-UV region Limited to ligands that do not strongly absorb in the Far-UV region Not possible with cloudy or colloid samples	1 mg/mL in a volume of ~25 μL when using a 0.1 mm cuvette
**Fluorescence Dye-based Differential Scanning Fluorimetry Assay (DSF)**		
Simple and fast to set up Uses low concentration/amounts of sample Screening of buffers and ligands that stabilise the protein Assessing the effects of mutations on the protein Monitoring protein–protein interactions	Requires a fluorescent dye Interactions of ligands with the dye may occur Requires cysteines embedded in the interior of the protein if CPM dye is used Removal of reducing agents from the purified protein if using CPM dye is required	Minimum: 1 to 15 µg of protein in a volume of 50 µL reaction
**Mid-Infrared Spectroscopy (MIR)**		
Simple and fast to set up Uses low concentration/volume of sample Measurement is independent of protein entity	Low concentration range for accurate measurement possibly requiring sample dilution Water and certain buffers (containing amide or amino functional groups, e.g., Tris) could interfere with the signal at the amide I band Standard curves need to be generated for each lipid/detergent for accurate measurement of their concentration	Protein range: 0.25 to 5.0 mg/mL Lipid/detergent range: 0.25 to 4.0 % Requires a sample volume of 2 µL
**Lipidic Cubic Phase Fluorescence Recovery After Photobleaching (LCP-FRAP)**		
Fast identification of ideal lipid/conditions prior to setting up large scale trials saving time and cost Low sample usage as protein concentration can be much lower than standard crystallisation concentration	Requires protein labelling with a fluorescent dye Incompatible with protein in Tris buffer if the Cy3-mono N-hydroxylsuccinimidyl (NHS) ester dye is used The sample must be kept in the dark as much as possible to prevent premature photobleaching	Minimum: 1 mg/mL of labelled protein reconstituted to LCP

## Abbreviations

Cy3	5, 5′-disulfato-1′-ethyl-3, 3, 3, 3- tetramethylindocarbocyanine
C_8_E_5_	Pentaethylene glycol monooctyl ether
C_12_E_8_	Dodecyl octaethylene glycol ether
CHS	Cholesteryl hemisuccinate
CMC	Critical micelle concentration
CPM	N-[4-(7-diethylamino-4-methyl-3-coumarinyl)phenyl]maleimide
Cryo-EM	Cryo-electron microscopy
Cymal-6	6-Cyclohexyl-1-Hexyl-β-d-Maltoside
DDM	n-Dodecyl β-d-maltoside
DLS	Dynamic light scattering
DM	n-Decyl-β-d-Maltoside
- (dRFU)/dT	Negative first derivative of the relative fluorescence units with respect to temperature
DSF	Differential scanning fluorimetry
GPCR	G-protein-coupled receptors
HTCD	High-throughput circular dichroism
IR	Infrared radiation
LALS	Low angle light scattering
LCP-FRAP	Lipidic cubic phase fuorescence recovery after photobleaching
LDAO	Lauryldimethylamine-N-oxide
LMNG	Lauryl maltose neopentyl glycol
LS	Light scattering
MIR	Mid-infrared spectroscopy
NBEs	New biological entities
NMEs	New molecular entities
NMR	Nuclear magnetic resonance
OG	n-Octyl-β-d-Glucoside
PDB	Protein Data Bank
PDC	Protein detergent complexes
Phe	Phenylalanine
PTFE	Polytetrafluoroethylene
RALS	Right angle light scattering
RI	Refracting index
RT-PCR	Real time polymerase chain reaction
SAXS	Small angle X-ray scattering
SDS-PAGE	Sodium dodecyl sulphate polyacrylamide gel electrophoresis
SEC	Size-exclusion chromatography
SEC-MALS	Size-exclusion chromatography multi-angle light scattering
SMALPS	poly(styrene-co-maleic acid) lipid particles
SMILPS	poly(styrene-co-maleimide) lipid particles
Trp	Tryptophan
Tyr	Tyrosine
UDM	n-Undecyl-β-d-Maltopyranoside
SRCD	Synchrotron radiation circular dichroism
UV	Ultraviolet
